# A novel method for quantitating delayed enhancement of the left atrium

**DOI:** 10.1186/1532-429X-13-S1-P248

**Published:** 2011-02-02

**Authors:** Christian B Moyer, Loren P Budge, Patrick T Norton, Christopher M Kramer, John D Ferguson, Jeffrey W Holmes

**Affiliations:** 1 University of Virginia, Charlottesville, VA, USA; 2 University of Virginia Health System, Charlottesville, VA, USA

## Introduction

Delayed contrast enhancement of the left atrium (DE-LA) in patients with atrial fibrillation (AF) has been reported to correlate well with outcomes after catheter ablation of AF. Currently utilized methods for DE-LA assessment require intensive post-processing and significant user-defined thresholding to obtain meaningful results.

## Purpose

We aimed to develop an accurate, reproducible method of DE-LA quantitation requiring less intensive post-processing and decreased user input, and to test this method in a typical AF population undergoing CMR.

## Methods

16 Patients with AF (7 with prior ablation, 9 with no history of ablation; 11 with paroxysmal AF, 5 with persistent AF) underwent CMR on a 1.5-T Siemens Avanto scanner. DE-LA images were acquired 15 min after contrast injection using a 3D inversion recovery, respiration-navigated, fat suppressed, ECG-gated, gradient echo pulse sequence, with acquisition parameters as follows: voxel size 1.2 x 1.2 x 2.5 mm, field of view: 30 x 30 cm, TR/TE: 4.3/2.4 ms, flip angle: 15°, bandwidth: 360 Hz/pixel, inversion time (TI) is based on a TI scout (range: 250-350 ms), with an average scan time of 5-9 minutes. To quantify DE-LA, we manually segmented the epicardial border of the left atrium, then extrapolated radially inward at a constant thickness (3 pixels) to define the endocardial border. Pixels within the contoured regions were tabulated into a pixel intensity histogram comprising the entire LA wall volume. We assumed the total distribution was composed of two underlying parts: normal and enhanced tissue. To separate these overlapping distributions, we fitted a mixture model containing two continuous Gaussian distributions, weighted by their respective areas. Using MATLAB, an intensity threshold was defined automatically at the intersection of the two distributions. The volume of enhancement isolated by the automatic threshold was then calculated and normalized to the total LA wall volume. Results are reported as a percentage of LA wall volume, and significant differences were determined using unpaired t-tests.

## Results

**Figure 1:**
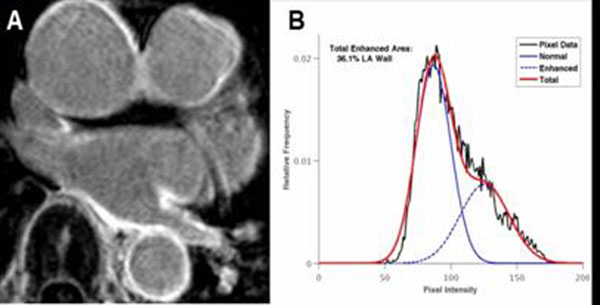
A representative DE-LA sample from a patient with prior ablation. **A** Axial DE-LA image showing enhancement of the left inferior pulmonary vein and posterior left atrial wall. **B** A pixel histogram of this patient, demonstrating two distinct pixel populations within the left atrial wall.

DE-LA was higher in patients with a previous AF ablation compared to patients with no prior left atrial ablation (19.8% vs. 9.5%, p<0.04). No difference in DE-LA was seen between paroxysmal and persistent AF (14.7% vs. 12.6%, respectively. p=NS). Figure 1.

## Conclusions

We have developed a novel method for quantitating DE-LA, which requires decreased user input compared to previously published methods. Using this method, we have shown a significant increase in DE-LA in patients who have had past ablation of AF compared to those without prior ablation.

